# COVID-19 Infection in Spinal Muscular Atrophy Associated with Multisystem Inflammatory Syndrome

**DOI:** 10.1155/2021/5862444

**Published:** 2021-06-30

**Authors:** Rafat Mosalli, Amirah Al Matrafi, Mohammed A. Ghazi, Gamal A. Aboumousatafa, Bosco Paes

**Affiliations:** ^1^Department of Pediatrics, Umm Al-Qura University, Mecca, Saudi Arabia; ^2^Department of Pediatrics, International Extended Care Center, Jeddah, Saudi Arabia; ^3^College of Medicine, Umm Al-Qura University, Mecca, Saudi Arabia; ^4^Pediatrics Critical Care, Department of Pediatrics, King Abdulaziz University, Jeddah, Saudi Arabia; ^5^Department of Pediatrics (Neonatal Division), McMaster University, Hamilton, ON, Canada

## Abstract

The coronavirus disease-2019 (COVID-19) is usually less severe and less prevalent in the pediatric population. Children with preexisting conditions such as neuromuscular impairments and chronic lung disease are more susceptible to COVID-19 and may incur several complications with a poor outcome. We present a case report of a 3-year-old-female with generalized hypotonia and respiratory failure due to spinal muscular atrophy who tested positive for COVID-19 and developed multisystem inflammatory syndrome that was treated with intravenous immunoglobulin and tocilizumab and subsequently died. The report highlights the importance of close surveillance, the use of protective measures during hospital visits, early testing, and diagnosis of COVID-19 in children with neurological disorders.

## 1. Introduction

The coronavirus disease-2019 (COVID-19) pandemic is caused by a novel severe acute respiratory syndrome coronavirus 2 (SARS-CoV-2) and is known to be less severe and less prevalent in children, as reported by Jin et al. [1]. At present, specific reasons for the low prevalence of the disease in children remain undefined. Nonetheless, Gurwitz et al. suggest that angiotensin-converting enzyme II (ACE2) acts as a binding site for COVID-19 and that ACE2 receptors are less mature in children [2], while Zimmermann and Curtis [3] postulate that the age gradient in children aligns with more robust innate immunity and changes in endothelial and clotting function. The World Health Organization (WHO) has classified COVID-19 severity into categories of mild illness, severe pneumonia, pediatric acute respiratory distress syndrome (PARDS), and sepsis [4]. Severe pneumonia in the pediatric population may manifest by the inability to breastfeed or drink, lethargy, unconsciousness, or convulsions. The child can present acutely with severe respiratory distress or as PARDS accompanied by hypoxemia with peripheral oxygen saturation level less than 90% [5]. Ventilator-dependent children are particularly susceptible to severe COVID-19 infection with associated complications. Neuromuscular and chronic lung diseases comprise these high-risk groups who require continuous use of ventilatory support with or without a tracheostomy and may quickly deteriorate with a high mortality rate [6].

Multisystem inflammatory syndrome in children (MIS-C) is a more serious evolution of COVID-19 that manifests as an acute febrile illness with gastrointestinal symptoms and causes widespread inflammation resulting in hypotension, toxic shock, and multiorgan failure [7–9]. The outpouring of cytokines at the nadir of the acute phase has been described as a storm associated with PARDS that correlates with disease severity, morbidity, and mortality if not treated expeditiously [10]. The features of MIS-C are akin to those of Kawasaki disease with mucocutaneous involvement, cardiac complications that comprise arrhythmias, acute heart failure, coronary abnormalities, and myocardial and left ventricular dysfunction [11–13]. More recently, it has been recognized that although the pathogenesis of both diseases is similar, MIS-C with PARDS more commonly afflicts older children (median age, 9 years) with greater elevations in inflammatory biomarkers [14].

## 2. Case Presentation

**Figure 1:**
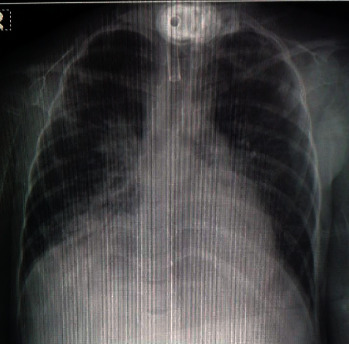
Chest X-ray prior to COVID-19: clear lung fields bilaterally with minimal veiling of the right middle lobe, tracheostomy in situ.

**Figure 2:**
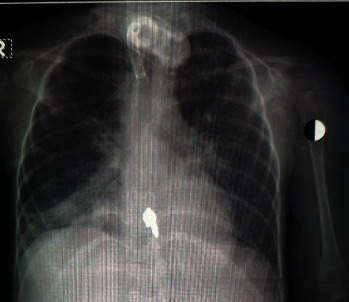
Onset of COVID-19: similar findings as in Figure 1 with exaggerated bronchovascular markings.

A three-year-old female child, who was a known case of spinal muscular atrophy (SMA) type one, was ventilator dependent via a tracheostomy and received feeding through a percutaneous gastrostomy tube. The patient was hospitalized from one year of age for extended supportive care because of recurrent episodes of lower respiratory tract infection secondary to repetitive aspiration. Her medical course was otherwise stable until the onset of high spikes of fever associated with tachycardia, increased respiratory secretions, feeding intolerance, abdominal distension, and vomiting. A septic screen was performed. The initial chest X-rays showed clear lung fields bilaterally with minimal veiling of the right middle lobe (Figures 1 and 2). Over the next 2 days, the fever persisted with associated tachycardia. A nasopharyngeal aspirate was sent for virology and a COVID-19 screen was ordered as part of the viral panel. A complete blood count showed a normal hemoglobin and leukocyte count with severe lymphopenia (2% of the total white cell count). The initial biochemistry and coagulation profile were in the normal range with a raised C-reactive protein (19.8 mg/L) and erythrocyte sedimentation rate (123 mm/hr). The blood culture showed no growth, and the respiratory secretions grew *Pseudomonas aeruginosa*. Antibiotics (piperacillin/tazobactam and gentamicin) were started, and the patient developed further abdominal distension and vomiting. No signs or symptoms of hepatic involvement were evident, and liver function tests at this stage were in the normal range. Of note, no mucocutaneous changes were evident.

Due to the persistent fever, gastrointestinal symptoms, and deterioration in respiratory status secondary to PARDS, a possible diagnosis of COVID-19 was entertained. The patient was commenced on antiviral therapy (oseltamivir), oral dexamethasone, and intravenous immunoglobulin (2 gm/kg over 12 hours) as recommended by the Saudi Ministry of Health guideline for suspected or confirmed cases of COVID-19 [15]. Other inflammatory/cytokine release markers of multisystem inflammatory syndrome in children (MIS-C) COVID-19 subsequently became evident (Table 1: high lactate dehydrogenase (353 U/L), C-reactive protein (318 mg/ml); D-dimer (1.5 *μ*g/ml), and serum ferritin (916 ng/ml). The initial COVID-19 culture was negative. Despite antiviral and dexamethasone therapy, the patient worsened with an altered level of consciousness (Glasgow coma scale 5/15) and developed septic shock with acute renal and multiorgan failure.

**Figure 3:**
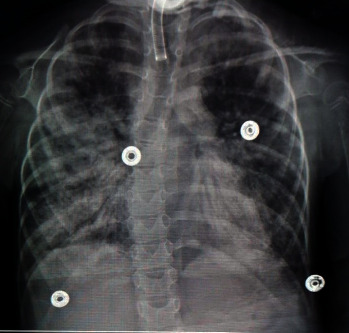
Day 7 of COVID-19: “whiteout” of the lungs bilaterally consistent with established acute respiratory distress syndrome (ARDS).

Closer questioning of the family about recent contact with COVID-19 indicated that family members who had recently visited the child were found to be COVID-19 positive. Therefore, a repeat COVID-19 culture was performed on the child and was positive. Additional investigations and biomarkers for COVID-19 were requested and showed a 1.3-to-16-fold increase in the C-reactive protein, erythrocyte sedimentation rate, lactate dehydrogenase, and ferritin levels (Table 1). Further treatment included the use of tocilizumab 8 mg/kg (interleukin-6 inhibitor) with a repeat dose in 12 hours based on a suspected diagnosis of cytokine release syndrome (CRS). Although ventilation, cardiac, and anti-inflammatory support was maximized, the patient continued to deteriorate with established PARDS (Figure 3), hemodynamic instability, and irreversible multiorgan failure and expired on the seventh day after multiple unsuccessful attempts at cardiorespiratory resuscitation. To the best of our knowledge, this is the first case of SMA type 1 with COVID-19-associated MIS-C to be reported in Saudi Arabia.

## 3. Discussion

SMA is a rare autosomal recessive disorder that involves a mutation in the survival motor neuron 1 gene and clinically manifests as hypotonia and progressive symmetrical muscle weakness resulting in failure to thrive and abnormal pulmonary function. Finkel et al. reported that SMA patients are more susceptible to respiratory infections and its related complications compared to healthy individuals [6].

In contrast to adults, pediatric patients infected with COVID-19 have milder symptoms, a better prognosis, and lower mortality rates. However, younger children with underlying long-term medical conditions are at far greater risk of developing PARDS with attendant complications that result in a longer duration of hospitalization and overall higher mortality rates [16, 17]. A systematic review by Montalvan et al. indicates emerging new evidence of neuroinvasion and dissemination to the central and peripheral nervous system resulting in worsening of preexisting neurological diseases [18]. Additionally, as in our case, new onset of neurological symptoms such as headaches, seizures, modified levels of consciousness, weakness, and hyposmia may become evident through viral neurotropism involvement of the SARS-CoV-2 receptor expressed in the nervous system and indirectly by hyperinflammatory and hypercoagulative states or ventilator-associated impairment [18, 19]. Thus, patients with SMA who are infected with COVID-19 are considered extremely high risk with a higher incidence of complications, a poor prognosis, and associated mortality [20].

One of the of COVID-19 complications is MIS-C. The WHO defined this condition based on the clinical presentation, elevated levels of inflammatory markers, documented infection, or contact with patients who have COVID-19 and elimination of other obvious causes of inflammation [21]. The criteria for MIS-C in association with COVID-19 as defined by the WHO [21], the United States Centers for Disease Control and Prevention [22], and the Royal College of Paediatrics and Child Health [23] include (1) fever >38°C for ≥ 24 hours; (2) laboratory evidence of inflammation such as an elevated C-reactive protein, erythrocyte sedimentation rate, fibrinogen, procalcitonin, D-dimer, ferritin, lactate dehydrogenase, or interleukin-6 and raised neutrophils and reduced lymphocytes; (3) multisystem with two or more organs involved (cardiac, renal, respiratory, neurological, and gastrointestinal); and (4) SARS-COV2 polymerase chain reaction or antigen positivity combined with exposure to COVID-19 within one week of disease onset.

In three systematic reviews and meta-analyses of MIS-C, the pulmonary radiological features were described as infiltrates or opacities which were reported by Yasuhara et al. among 131 subjects (38.3% [95% CI, 29.7–46.9]) [24]. Badaradan et al. [25] found across 8 studies that 45.9% (95% CI, 34.1%–58.2%) of the patients had abnormalities on chest X-ray or computed tomography which included ground-glass opacity, interstitial abnormalities, or local patchy shadowing. Kaushik et al. [26] found similar imaging abnormalities in 90 (13.7%) of the patients, which resembled the findings in our case. A complete blood count tends to show elevated neutrophils and reduced lymphocytes which were replicated in our findings [24–26]. The laboratory findings in MIS-C uniformly indicate ≥4 elevated inflammatory markers such as erythrocyte sedimentation rate, C-reactive protein, procalcitonin, and ferritin, with a simultaneous rise in the International Normalized Ratio, fibrinogen, D-dimer, alanine aminotransferase, lactate dehydrogenase, interleukin-6 and interleukin-8, and cardiac markers (B-type natriuretic peptide, N-terminal proB-type natriuretic peptide, and troponin) [14, 27–29]. It should be noted that elevated C-reactive protein, lactic dehydrogenase, neutrophil-to-lymphocyte ratio, and lower platelet counts (<150 × 10^3^ *μ*L) are associated with more severe disease [30] and that there are some similarities between the clinical presentation of MISC-C and Kawasaki disease that could delay the diagnosis as reported by Licciardi et al. [31].

The laboratory findings of CRS are lymphopenia and a rise in ferritin, C-reactive protein, creatinine, liver enzymes, and lactate dehydrogenase. A cytokine storm is defined as a severe immunological response resulting in a rapid release of cytokines (IFN-*γ* and TNF-*α*), interleukins (IL-1, IL-2, IL-6, IL-10, and IL-18), and other immune mediators [10, 32, 33]. A variety of signs and symptoms have been reported as part of CRS ranging from nausea and fever to shock [34, 35]. Our patient had the clinical hallmarks of CRS which included hemodynamic instability, life-threatening shock, multiorgan failure, hyperferritinemia, and leucopenia [32, 34, 35], but cytokine and interleukin biomarkers were not assessed because of the rapid deterioration in clinical status. The short symptom duration as noted in our case is associated with poor outcome, need for extracorporeal membrane oxygenation, and death [34]. Our patient met the diagnostic criteria for MIS-C based on Tier 1 and Tier 2 testing [35] and CRS, but it is important to note that the temporal association with COVID-19 was shorter than usual and it is difficult to differentiate between the two entities which overlap in clinical symptomatology and diagnostic criteria.

The COVID-19 management protocol and guidelines delineated by the Saudi Ministry of Health were followed in the care of this patient who received intravenous immunoglobulin, tocilizumab, and mechanical ventilation [15]. The use of cytokine inhibitors tocilizumab, anakinra, and baricitinib is a potential option for the treatment of severe COVID-19 [33], but none of them have been investigated in prospective clinical trials in the pediatric population. In five reviews among patients with MIS-C, the following therapies were employed: intravenous immunoglobulin (63–100%), corticosteroids (49–70%), anakinra (8–13%), tocilizumab (6.5–27%), remdesivir (3–8%), infliximab (1.2–8%), and plasma (*n* = 3) [24, 26, 27, 29, 36–38]. Intravenous immunoglobulin combined with methylprednisolone compared to intravenous immunoglobulin alone is significantly effective in reducing fever, the need for second-line drug therapy, hemodynamic support, and length of intensive care stay [39]. Immunoglobulin and steroids are the mainstay of MIS-C therapy, while recombinant human IL-1 receptor antagonists (anakinra) and IL-6 neutralizing agents (tocilizumab) should be reserved for critically ill patients with shock, PARDS, and hyperinflammation [35].

Comorbidities in children pose a higher risk for serious COVID-19 disease, mechanical ventilation, and resultant death [20, 40]. LaRovere et al. reported that children hospitalized with COVID-19 or MIS-C who developed neurologic involvement were more likely to have preexisting neurologic disorders including seizures, neuromuscular conditions, autism, or developmental delay (22%) compared with those without (8%) [41]. Shekerdemian et al. [17] noted that 40% of patients admitted to intensive care with COVID-19 had developmental delay, a genetic anomaly, or were technologically dependent on respiratory support. Most of the patients (96%) both with and without neurologic involvement usually survive [41]. MIS-C is an uncommon yet serious complication of COVID-19 infection in patients with SMA, since baseline muscle weakness is exacerbated during respiratory infections resulting in acute respiratory decompensation [6, 42]. PARDS in SMA types 1 and 2, coupled with CRS may further enhance both morbidity and mortality. Unlike our case, current trends indicate that majority of patients with MIS-C survive despite life-threatening illness and the overall mortality rate ranges from 0 to 3% dependent on the duration of symptoms and the severity of illness [14, 24–30, 34, 36–38].

## 4. Conclusions

MIS-C has emerged as one of the life-threatening complications of COVID-19. Due to the absence of clinical trials in the pediatric population, there is no generalized consensus about which medication results in favorable outcomes, but an initial combined therapeutic modality involving steroids and immunoglobulin indicates improved survival. Children with neurological impairments such as SMA comprise a high-risk category for COVID-19. The high morbidity and potential mortality incurred among this cohort should be drivers for heightened surveillance, and stringent personal protective measures and social distancing should be adopted when parents and families visit children hospitalized with medical complexity.

## Figures and Tables

**Table 1:** Investigations before and during COVID-19 course of illness.

Tests	Reference range	Before COVID-19	COVID-19 suspected	COVID-19 identified	During COVID-19 course
*CBC*					
Hemoglobin	10.5–14.0 g/dL	10.7	9.4	7.7	9.7
WBCs	5–15 × 10^3^ *µ*L	16.6	9.6	31.6	86.1
Lymphocytes	15–80% of WBC count	12.7%	8.9%	5.4%	2.0%
Platelets	150–400 × 10^3^ *µ*L	474	270	529	811

*Biochemistry*					
Creatinine	55–110 *µ*mol/l	12	6	11	6
LDH	135–225 U/L	—	353	363	464

*Coagulation*					
INR	1-2	1.02	0.88	1.1	1.15
PT	11–14 sec	13.8	12	14.8	15.4
PTT	30–40 sec	37.5	31	26.3	22.9
D-dimer	0–0.55 *µ*g/ml	—	1.5	1.3	1.5

*Inflammatory markers*					
CRP	0–5 mg/L	19.8	37.2	318	219.7
ESR	0–10 mm/hr	123	126	280	150
Ferritin	13–400 ng/ml	—	917	1732	2000

*Cultures*					
Viral panel	—	Negative	Negative	Negative	Negative
COVID-19	—	—	Negative	Positive	Positive

CBC, complete blood count; CRP, C-reactive protein; COVID-19, coronavirus disease-2019; ESR, erythrocyte sedimentation rate; INR, International Normalized Ratio; LDH, lactate dehydrogenase; PT, prothrombin time; PTT, partial thromboplastin time; WBC, total white blood cells.

## Data Availability

Data of this case report are available upon request.
